# *Dirofilaria immitis* and *Dirofilaria repens* in mosquitoes from Corsica Island, France

**DOI:** 10.1186/s13071-021-04931-y

**Published:** 2021-08-26

**Authors:** Laidoudi Younes, Hélène Barré-Cardi, Samia Bedjaoui, Nazli Ayhan, Marie Varloud, Oleg Mediannikov, Domenico Otranto, Bernard Davoust

**Affiliations:** 1 Aix Marseille Univ, IRD, AP-HM, MEPHI, Marseille, France; 2 IHU Méditerranée Infection, Marseille, France; 3 Department of Veterinary Medicine, University of Bari, Valenzano, Bari, Italy; 4 OCIC, Office de l’Environnement de la Corse, Corte, France; 5 Laboratory of Food Hygiene and Quality Insurance System (HASAQ), Higher National Veterinary School, Issad Abbes, Oued Smar, Algiers, Algeria; 6 Aix Marseille Univ, IRD 190, INSERM U1207, Unité des Virus Emergents, Marseille, France; 7 Ceva Santé Animale, 10, Av de la Ballastière, Libourne, France; 8 Faculty of Veterinary Sciences, Bu-Ali Sina University, Hamedan, Iran; 9 Animal Epidemiology Experts Group of the Military Health Service, Tours, France

**Keywords:** *Dirofilaria immitis*, *Dirofilaria repens*, Mosquitoes, Transmission suitability, Corsica

## Abstract

**Background:**

*Dirofilaria immitis* and *Dirofilaria repens* are the main causative agents of heartworm disease and subcutaneous dirofilariasis in domestic and wild canids, respectively. Both pathogens have zoonotic potential and are transmitted by mosquitoes. The present study aimed to determine the transmission period, prevalence and diversity of *Dirofilaria* spp. vectors from endemic areas of Corsica (France).

**Methods:**

A monthly point data model based on average temperature recorded by four meteorological stations during 2017 was used to calculate the *Dirofilaria* transmission period. From June to September 2017, female mosquitoes (*n* = 1802) were captured using Biogents^®^ Sentinel 2 traps lured with carbon dioxide and BG-Lure™ or octanol. Mosquitoes were identified to species level, pooled accordingly, and screened using multiplex real-time qPCR to detect *D. immitis* and *D. repens*.

**Results:**

The monthly point data model showed the possible transmission of *Dirofilaria* spp. from the third week in May to the last week in October in the studied area. Mosquitoes were identified as *Ochlerotatus caspius* (*n* = 1432), *Aedes albopictus* (*n* = 199), *Culex pipiens* sensu lato (*n* = 165) and *Aedes vexans* (*n* = 6) and were grouped into 109 pools (from 1 to 27 specimens, mean 11.4 ± 0.7), of which 16 scored positive for *Dirofilaria* spp. (i.e., *n* = 13; estimated infection rate [EIR] = 1.1% for *D. immitis* and *n* = 3; EIR = 0.2% for *D. repens*). Specifically, 6 (i.e., EIR = 3.8%) of 15 pools of *Ae. albopictus* were positive for *D. immitis*, 2 of 14 of *Cx. pipiens* s.l. were positive for *D. immitis* and *D. repens*, respectively, and 8 of 77 pools of *Oc. caspius* were positive for *D. immitis* (i.e., *n* = 6; EIR = 0.4%) and *D. repens* (i.e., 2; EIR = 0.1%). The highest mosquito infection rate was recorded in July (EIR = 2.5%), then in June (EIR = 1.3%) and September (EIR = 0.6%).

**Conclusions:**

The data suggest that both *Dirofilaria *species are endemic and occur possibly in sympatry in the studied area in Corsica, highlighting the need to implement preventive chemoprophylaxis and vector control strategies to reduce the risk of these filarioids in dog and human populations.

**Graphical Abstract:**

**Figure:**
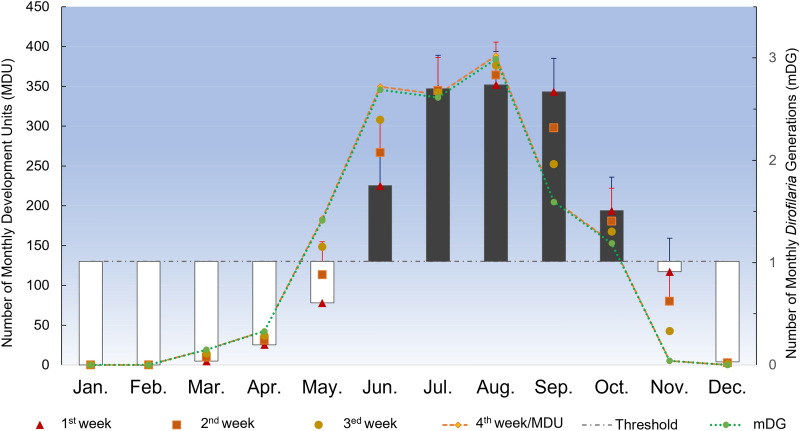

## Background

*Dirofilaria immitis* and *Dirofilaria repens* are zoonotic filarioid nematodes responsible for canine cardiopulmonary and subcutaneous dirofilariasis, respectively [1]. *Dirofilaria immitis* is of great veterinary importance while *D. repens* is the main causative agent of human dirofilariasis in the old world [2]. These mosquito-borne filarioids share the same definitive hosts (mostly canids), and several mosquitoes species (i.e., mosquitoes of the genera *Culex*, *Aedes*, *Ochlerotatus*, *Anopheles*, *Coquillettidia*, *Armigeres*, *Mansonia* and *Psorophora*) have been reported as competent vectors [3]. Several of these vectors feed indiscriminately on dogs and humans, resulting in a zoonotic sympatric occurrence in endemic areas [2].

Molecular detection of filarioid parasites from the bloodsucking arthropods is one of the most effective strategies for assessing the prevalence of vectors and/or pathogens in a given area. For example, to assess the prevalence of canine filarioids, a molecular-based approach was recently proposed for the diagnostic and xenomonitoring of skin- and blood-associated microfilariae from dog ticks [4]. Therefore, two duplex real-time polymerase chain reaction (PCR) assays have been standardized for the xenomonitoring of *D. immitis* and *D. repens* in mosquito vectors [5, 6]. Previous molecular xenomonitoring studies revealed the presence of at least three filarioid nematodes (i.e., *D. immitis*, *D. repens* and *Setaria tundra*) in several mosquito species in Europe, mostly belonging to the genera *Aedes*, *Anopheles*, *Coquillettidia*, *Culex*, *Culiseta* and *Ochlerotatus* [5–22].

Canine dirofilariasis is endemic in Southern Europe [23–26]. Corsica, a French island in the Mediterranean basin, is known as an epidemiological hotspot from which several cases of vector-borne diseases in humans and dogs are imported to the mainland. This is the case of *D. immitis* in dogs from Corsica [27, 28] and human dirofilariasis caused by *D. repens* in visitors to the island [29]. Previous epidemiological studies confirmed the circulation of *Dirofilaria* spp. in human, dog and mosquito (i.e., *Ae. albopictus*) populations from the island [6, 30, 31]. However, data on the seasonality of transmission, diversity of the *Dirofilaria* vector and prevalence are sparse. This work advances understanding of the transmission period, prevalence and potential vectors of *Dirofilaria* spp. from endemic areas of Corsica (France).

## Methods

### Study area and seasonal transmission of *Dirofilaria* spp.

Monthly point data (monthly average temperatures) recorded in 2017 from four meteorological stations (i.e., Aleria: 42°06′53″N, 9°30′48″E; Solenzara: 41°55′36″N, 9°24′19″ E; Borgo: 42°33′17″N, 9°25′41″E and Solaro: 41°54′17″N, 9°19′37″E) were obtained from https://www.prevision-meteo.ch/ and were processed as described elsewhere [32]. Briefly, mean monthly and weekly development units (DU) for *Dirofilaria* spp. were calculated using the monthly point data model by Cuervo et al. [32] for *D. immitis*. The DUs represented the predicted degrees Celsius above the threshold of 14 °C, as described for *D. immitis* [33] and *D. repens* [34]. Mean monthly development units (mMDU) were calculated as mMDU = [mean monthly temperature–14]*30 for each month. Monthly *Dirofilaria* generations (mDG) were calculated by dividing the number of mMDU by the threshold of 130 DU required for extrinsic development of one generation of microfilariae (L1) to infective larvae (L3) within the vector [34]. To determine the initial transmission period, weekly development units (WDU) were calculated for each week of each month as described elsewhere [32].

### Mosquito sampling and processing

In June, July and September 2017, female mosquitoes (*n* = 1802) were captured from four sites (i.e., Aleria, Solenzara, Solaro and Borgo) in the department of Haute-Corse, Corsica, France. Mosquito capture was performed using Biogents^®^ Sentinel 2 (*n* = 16) (Biogents AG, Regensburg, Germany) traps lured with carbon dioxide and BG-Lure™ (Biogents AG, Regensburg, Germany) or octanol (Biogents AG, Regensburg, Germany) in Aleria (*n* = 4), Solenzara (*n* = 4), Solaro (*n* = 6) and Borgo (*n* = 2). At each time period, traps were placed approximately 1.5 m above ground and were installed at 17:00 and recovered 4 days later around 10:00. Mosquitoes were individually identified to species level using morphological keys [35] and then pooled (from 1 to 27 specimens, mean 11.4 ± 0.7) by species, sampling dates and province.

One hundred and nine pooled (from 1 to 27, mean 11.4 ± 0.7) female specimens were prepared. For each mosquito pool, a 10-min bead-based mechanical lysis was performed in the TissueLyser apparatus in the presence of 800 µl of MEM medium (Sigma Aldrich). Mosquito lysates were centrifuged at 13,000*g* for 3 min, and genomic DNA was extracted from 200 µl of the supernatant in the presence of 100 µl of lysis buffer. Extraction was performed using the QIAcube kit (Qiagen, Courtaboeuf, France) according to the manufacturer’s instructions. DNA was eluted in a final volume of 100 µl and stored at −20 °C until analysis. Finally, genomic DNA was analysed for the presence of *Dirofilaria* spp. using a multiplex real-time qPCR assay as described elsewhere [36].

### Data analysis

Differences in *Dirofilaria* spp. infection were evaluated between mosquito species, sampling period and province using the analysis of covariance (ANCOVA) model within XLSTAT software (Addinsoft, Paris, France, 2018). The minimum infection rate (MIR) [37] and the estimated infection rate (EIR) [38] were calculated using the following formulas: MIR = (*x*/*n*)*100 and ERI = (1 − (1 − *x*/*m*)^1/*k*^)*100, where *x* is the number of positive pools, *n* is the total number of mosquitoes tested, *m* is the number of mosquito pools and *k* is the average number of mosquitoes in each pool.

## Results

**Fig. 1:**
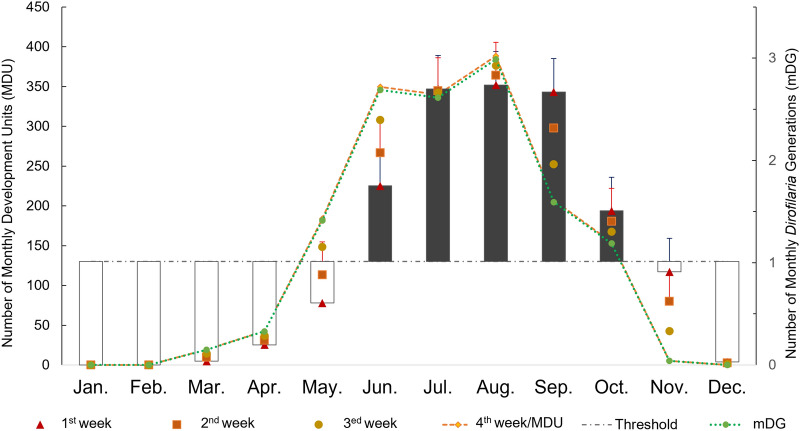
An open-high-low-close chart showing the intensity and the time frame of the extrinsic incubation of *Dirofilaria *spp. within the vector in the studied area.  Bars indicate the extinct (white) and active (grey) transmission period. Transmission intensity is represented by both generations (DG) and development units (DU) of *Dirofilaria *spp. on a weekly schedule. The fourth WDU of each month was considered equivalent to the corresponding MDU

**Fig. 2:**
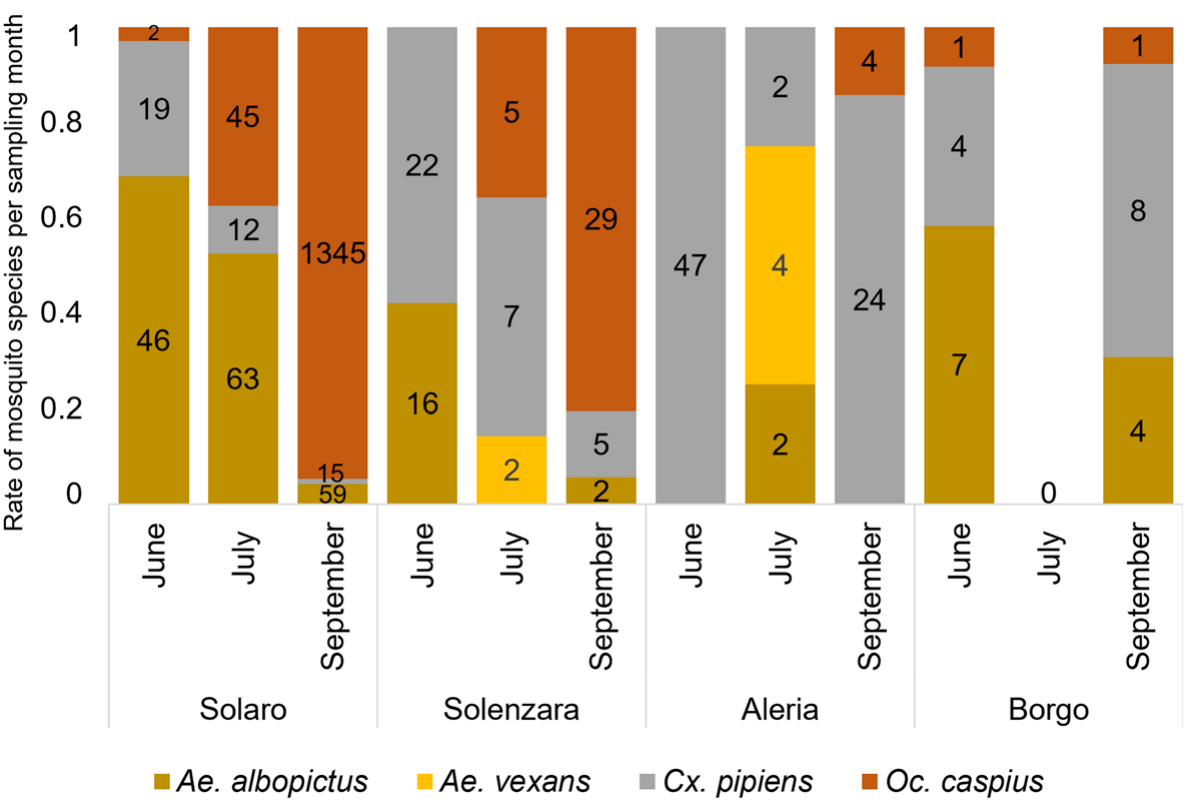
Distribution of female mosquitoes according to their species, sampling month and province

The monthly point data model indicated the potential transmission of *Dirofilaria* spp. for 22 weeks (from the third week in May to the last week in October) in the study area (Fig. 1). During the summer season (from July to September), up to 2.5 mDG were recorded each month with a maximum activity of 3 mDG in August (Fig. 1). Morphological identification of mosquitoes revealed the presence of at least four species, dominated by *Ochlerotatus caspius* (*n* = 1432; 79.8%) followed by *Aedes albopictus* (*n* = 199, 11.0%), *Culex pipiens* sensu lato (*n* = 165; 9.2%) and *Aedes vexans* (*n* = 6; 0.3%). Most mosquitoes were caught in Solaro province (*n* = 1606; 89.1%), where the mosquito population was dominated by *Oc. caspius* (*n* = 1392; 86.7%) and *Ae. albopictus* (*n* = 168, 10.5%). Mosquito abundance in the other provinces ranged from 25 specimens in Borgo to 83 and 88 specimens in Aleria and Solenzara, respectively. The highest number of mosquitoes (*n* = 1496; 81.5%) was caught during September (Fig. 2).

With the exception of the *Ae. vexans* pools, *Dirofilaria* spp. were detected in at least one mosquito pool per species with the highest infection rates for *D. immitis* in *Ae. albopictus* (MIR = 3%; EIR = 3.8%) and for *D. repens* in *Cx. pipiens* s.l. (MIR = 0.6%; EIR = 0.6%). *Dirofilaria immitis* was detected with the highest infection rates (MIR = 2.2%; EIR = 2.5%) in July, followed by June (MIR = 1.2%; EIR = 1.3%) and September (MIR = 0.5%; EIR = 0.6%). In contrast, *D. repens* was detected only in June (MIR = 0.6%; EIR = 0.6%) and September (MIR = 0.1%; EIR = 0.1%). *Dirofilaria* were identified in two provinces surveyed (i.e., *D. immitis* in Solenzara and both species in Solaro provinces; Table 1).

**Table 1:** Mosquito pools and their positivity for *Dirofilaria* spp. according to mosquito species, sampling month and province

Variables	Specimens (*n*; m; k; SE)	*Dirofilaria immitis*				*Dirofilaria repens*			
		Positive pools	MIR	EIR	*P*-value *P*-value^a^	Positive pools	MIR	EIR	*P*-value *P*-value^a^
Mosquito species									
* Ochlerotatus caspius*	(1432; 77; 18.6; 0.5)	6	0.4	0.4	ref.	2	0.1	0.1	ref.
* Aedes albopictus*	(199; 15; 13.3; 2.2)	6	3.0	3.8	0.0001	0	na	na	0.707
* Aedes vexans*	(6; 3; 2.0; 0.6)	0	na	na	0.757	0	na	na	0.566
* Culex pipiens*	(165; 14; 11.8; 2.1)	1	0.6	0.6	0.207	1	0.6	0.6	0.125
Sampling month									
September	(1496; 83; 18; 0.6)	8	0.5	0.6	ref.	2	0.1	0.1	ref.
July	(139; 12; 11.6; 2.3)	3	2.2	2.5	0.406	0	na	na	0.701
June	(167; 14; 11.9; 2.5)	2	1.2	1.3	0.829	1	0.6	0.6	0.197
Province									
Solaro	(1606; 83; 19.4; 0.3)	12	0.7	0.8	ref.	3	0.2	0.2	ref.
Aleria	(83; 9; 9.2; 3.1)	0	na	na	0.084	0	na	na	0.092
Solenzara	(88; 9; 9.8; 2.6)	1	1.1	1.2	0.211	0	na	na	0.232
Borgo	(25; 8; 3.1; 0.8)	0	na	na	0.025	0	na	na	0.192
Global infection rate	(1802; 109; 11.4; 0.7)	13	0.7	1.1	na	3	0.2	0.2	na
Statistics		ANCOVA: *R*^2^_(100)_ = 0.193				ANCOVA: *R*^2^_(100)_ = 0.064			
		ANCOVA: *F*_(8,108)_ = 2.993, *P*^b^ = 0.005				ANCOVA: *F*_(8,108)_ = 0.861, *P*^b^ = 0.552			

ANCOVA statistics are also reported along with the percentage of minimum infection rate (MIR) and estimated rate of infection (ERI)

*n*, number of mosquito specimens; *m*, number of mosquito pools; *k*, average number of specimens per pools; SE, standard error; ref, fixed reference group for ANCOVA analysis; na, not applicable

^a^*P*-value calculated with Student’s *t*-test within ANCOVA model for the effect of individual factors (i.e., mosquito species, sampling month and province) on *Dirofilaria* spp. infection

^b^*P*-value calculated with Fisher test within ANCOVA model for the global effect of grouped factors (i.e., mosquito species, sampling month and province) on *Dirofilaria* spp. infection

## Discussion

This study reports data on *Dirofilaria* spp. in mosquitoes collected in Corsica along with a prediction model to forecast the seasonal transmission of these filarioids, providing information about vector diversity and infestation rates with *Dirofilaria* spp. in Corsican mosquito species.

*Dirofilaria* transmission is related to an episystem complex involving several factors including temperature, vector and host abundance [2]. Data herein indicate that *Dirofilaria* transmission may occur over 22 weeks (from May to October), with maximum activity of 2.6 to 3 mDG during the summer period (June to August), as reported in the southern regions of Europe [1], especially in Italy [39]. Interestingly, in this study, the highest MIR/ERI were recorded from *Ae. albopictus* in July, which coincides with the first peak of seasonal transmission (more than 2.6 mDG). Moreover, during the highest transmission peaks (June and July), mosquito fauna was dominated by *Ae. albopictus* and *Cx. pipiens* s.l., which are well-known vectors for *Dirofilaria* spp., therefore representing an epidemiological risk for infection to dogs and humans. It is worth noting that those are the months when a large number of tourists visit the island, often along with their pets. The relationship between prediction and actual prevalence of *Dirofilaria* has already been confirmed in several studies [32, 40, 41]. However, this period could be extended by the presence of heat islands, microenvironments such as buildings and parking lots retaining heat during the day. Consequently, the extrinsic development of *Dirofilaria* larvae becomes possible during the cold season [2, 42, 43]. Furthermore, some *Dirofilaria* vectors such as *Cx. pipiens* s.l. are known to overwinter as mated females, which may lead to the quick development of *Dirofilaria* larvae with subsequent warming periods [42, 44]. Hence, in order to prevent *Dirofilaria* transmission in such areas, these factors have to be carefully considered when adopting chemoprophylaxis protocols, as already demonstrated in the field against *Dirofilaria* spp. infection in dogs [31].

The present data showed that Corsican mosquito fauna was dominated by *Oc. caspius* (*n* = 1432; 79.8%), which is in agreement with previous reports from southern Europe (Italy) [45, 46]. In addition to confirming the previous report on *Dirofilaria* spp. from *Ae. albopictus* [6], the present data show for the first time the presence of *Dirofilaria* spp. DNA in *Oc. caspius* and *Cx. pipiens* s.l. in France, as reported in previous European studies [5–22]. Despite the large natural infestation of European mosquitoes from endemic areas [5–22], only a few species have been experimentally confirmed as competent for *Dirofilaria* spp. (e.g., *Cx. pipiens* s.l., *Ae. albopictus*, *Aedes aegypti*, *Aedes japonicus*, *Aedes geniculatus* and *Aedes koreicus*) [47–50]. Under natural conditions, blood pathogens can be found in haematophagous arthropods after a blood meal, without implying that they act as vectors [4]. Therefore, the data presented here highlight the potential role of *Oc. caspius*, along with well-known vectors (i.e., *Cx. pipiens* s.l. and *Ae. albopictus*), in the transmission of *Dirofilaria* spp. to humans and animals in this tourist area. *Aedes albopictus* was found to be highly infested with *D. immitis* (MIR = 3%, EIR = 3.8%), as shown previously [6, 15, 51]. Both *Dirofilaria* spp. were detected in *Oc. caspius* from Solaro, an area endemic for dirofilariasis [6, 31]. It should be noted that this urban area is also characterized by a typical environment for mosquito development, together with the availability of definitive hosts (dogs) [31], which could explain the infestation of *Oc. caspius* with *Dirofilaria* spp. Despite the low *Dirofilaria* spp. infection rates of *Oc. caspius* (from 0.1 to 0.4%), this is the dominant species (86.7%) captured, therefore suggesting its possible role in their transmission [5, 52].

The highest infection rate with *D. immitis* and *D. repens* was detected in *Cx. pipiens* s.l. mosquitoes (MIR = 0.6%, EIR = 0.6%) followed by *Oc. caspius* (MIR and EIR ranged from 0.1 to 0.4%), as already documented in central European Russia, Germany, Italy, Turkey and the Republic of Belarus [53]. High positivity of *Cx. pipiens* s.l. for *D. immitis* and *D. repens* was demonstrated by both molecular and parasitological studies [54, 55]. Finally, the absence of *Dirofilaria* spp. DNA from *Ae. vexans* in the present study might be related to the smaller number of specimens examined (*n* = 6).

## Conclusions

The present study highlights the sympatric occurrence of *D. immitis* and *D. repens* as well as the epidemiological pressure exerted by the length of the transmission season and the diversity of *Dirofilaria* spp. vectors in Corsica. We highlight public health risks, as Corsica attracts more than 750,000 visitors and their pets each year, which could pose an important risk for the transmission and spread of these zoonotic mosquito-borne filarioids. Moreover, the development of specific assays able to identify the infested/infective mosquito species with *Dirofilaria* spp. are needed for an integrative surveillance approach.

## Data Availability

The data supporting the conclusions of this article are included within the article.

## Abbreviations

MIR: Minimum infection rate
EIR: Estimated infection rate
ANCOVA: Analysis of covariance
